# Effect of different pacing strategies on 4-km cycling time trial performance

**DOI:** 10.1590/1414-431X2022e12351

**Published:** 2023-01-09

**Authors:** V. Vieira-Cavalcante, L.P. Venancio-Dallan, O. Pereira-Santana, R. Bertuzzi, F. Tomazini, D.J. Bishop, G. Cristina-Souza, A.E. Lima-Silva

**Affiliations:** 1 Grupo de Pesquisa em Performance Humana, Universidade Tecnológica Federal do Paraná, Curitiba, PR, Brasil; 2 Grupo de Estudos em Desempenho Aeróbio, Universidade de São Paulo, São Paulo, SP, Brasil; 3 Institute for Health and Sport, Victoria University, Melbourne, Australia; 4 Grupo de Pesquisa em Exercício e Nutrição, Universidade do Estado de Minas Gerais, Passos, MG, Brasil

**Keywords:** All-out start strategy, Self-paced strategy, Even-paced strategy, Fast-start strategy, Brain regulation

## Abstract

In cycling, there is a body of evidence that supports that an all-out start strategy is superior to an even-pacing strategy, but it is unknown whether an all-out start strategy is superior to a self-paced strategy. In the present study, we investigated the effects of three different pacing strategies on 4-km cycling time trial performance. After preliminary trials (familiarization trials and a baseline 4-km cycling time trial), in a randomized and counterbalanced order, twelve male cyclists (32.3±7.2 years old, maximum rate of O_2_ uptake (V̇O_2_peak) 4.3±0.4 L/min) completed: 1) a self-paced 4-km cycling time trial; 2) an all-out start (∼10 s), followed by maintenance of the average baseline trial power for the first km and self-paced cycling for the remaining trial (all-out+mean); and 3) an all-out start (∼10 s), followed by a power 5% above the average baseline trial power for the first km and self-paced cycling for the remaining trial (all-out+5%mean). Although there was a significant interaction between power and distance (P=0.001) with different power distribution profiles throughout the trial, there was no significant difference (P=0.99) between the three strategies for overall exercise performance (self-paced 379.8±13.9 s, all-out+mean 380.0±16.0 s, and all-out+5%mean 380.2±11.5 s). Oxygen uptake, rating of perceived effort, and heart rate were also similar across the pacing strategies. Different all-out start strategies did not confer additional benefits to performance compared to a self-paced strategy.

## Introduction

The distribution of power output throughout a self-paced endurance exercise has been defined as pacing strategy and has a great influence on overall performance (1,2). Although pacing strategy is mostly self-determined, athletes might benefit from a “forced”, relatively high power start of a short-duration (e.g., 4-km) cycling time trial (2-4), which is performed in the severe-intensity domain (i.e., above critical power) (5). In fact, the adopted pace during the first quarter of a short-duration cycling task lasting ∼5 min can meaningfully influence finishing time, suggesting that the first quarter of a short-duration time trial is a crucial part determining overall performance (6,7). In this regard, some evidence suggests that an all-out start strategy lasting ∼10-15 s, followed by a quick transition to an even pace, significantly improves overall performance compared to an even or fast-start pacing strategy (6,8). However, to our knowledge, there is no research comparing different all-out start strategies against a self-paced strategy during short-duration events such as a 4-km cycling time trial.

An explanation for the improvement in overall performance when using an all-out start strategy is that increasing power to overcome the inertia of a stationary start minimizes the acceleration period, resulting in less time spent in suboptimal speed (9,10). Additionally, the rate of phosphocreatine (PCr) breakdown increases as a function of the exercise intensity within the severe-intensity domain (5). Because all-out start strategy increases starting power, it would be expected greater rates of PCr breakdown adopting this type of pacing strategy (8,11). In addition, as the products of PCr breakdown (creatine and inorganic phosphate) stimulate oxidative phosphorylation, muscle and pulmonary O_2_ uptake (V̇O_2_) response are directly proportional to the rate of PCr breakdown (12). Consequently, an all-out start strategy can speed up V̇O_2_ response via increased rate of PCr breakdown (8,13). This accelerated V̇O_2_ kinetics will result in increased total ATP available to fuel exercise, ultimately increasing overall performance (1,6-8).

However, some unresolved questions should be highlighted. A “forced” all-out start strategy has mostly been compared with an equally “forced” even-pace strategy (6,8) or “forced” fast-start strategy (7). In all these three pacing strategies, athletes do not employ their own pacing and there is no final sprint (6-8). It is interesting to note that when athletes are free to choose their own pacing during a short-duration cycling time trial, they spontaneously combine these strategies (14,15). For example, a 4-km cycling time trial is performed with a natural fast-start, followed by a gradual decline until a constant power and a further increase in power during a final sprint (14,15). These differences are important because a self-paced exercise might be less physically challenging than enforced pace exercise (16). In addition, current theoretical models have suggested that pacing strategy is a behavioral expression of a continuous decision-making process, in which the brain regulates how and when to invest energy based on knowledge of the endpoint, memory of prior events, and external (environmental) and internal (metabolic) feedbacks (17-20). This decision-making process assures that maximal rating of perceived effort (RPE) is achieved only at the endpoint of the trial (21,22). Thus, whether an all-out start strategy is still able to improve performance compared with a self-paced strategy is currently unknown, but it would be reasonable to hypothesize that an all-out strategy is not superior in this case because a self-paced strategy already presents a naturally regulated fast start (14,15). Thus, a naturally regulated fast start would attenuate the potential advantage of an all-out start strategy, but this hypothesis has not been tested yet. In addition, any remaining disadvantage of the self-paced strategy may be further compensated by a spontaneous final sprint, but this hypothesis has also not been tested. Thus, an experimental comparison between the all-out start strategy and self-paced strategy would determine whether it is advantageous to intentionally manipulate pacing strategy during short-duration cycling time trial events.

An additional practical factor is that the all-out period is immediately followed by maintenance at mean power for the remaining first quarter of the trial to prevent intramuscular disturbances (9,23). The faster V̇O_2_ kinetics during the all-out start strategy signaling an early increase in aerobic energy supply might preserve part of the anaerobic capacity (8,13). This preserved part of the anaerobic capacity may be better explored by employing a higher power immediately after the all-out phase (i.e., during the first quarter of the trial). In fact, changes in pacing strategy alters the rate of anaerobic energy expenditure (3,14). There is, however, no study manipulating the post all-out phase; therefore, it remains unclear whether athletes would be able to support a slightly higher power than the mean power after the all-out period without developing premature fatigue. It is reasonable to hypothesize that this would not compromise a final sprint, and overall performance would be maximized (24,25). Thus, knowing whether an adapted all-out start strategy is superior to a traditional all-out start strategy or a self-paced strategy might provide additional support to athletes and coaches in their choice of best pacing strategy.

In the present study, we investigated the effects of three different pacing strategies on 4-km cycling time trial performance. The three pacing strategies tested were: 1) a self-paced strategy; 2) an all-out start strategy for the first 10 s, with further maintenance at average power obtained in a baseline trial for the remaining first quarter (i.e., first km), and self-paced for the remaining 3 km (all-out+mean trial); and 3) an all-out start strategy for the first 10 s, with further maintenance of a 5% higher than average power obtained in the baseline trial for the remaining first km, and self-paced cycling for the remaining 3 km (all-out+5%mean trial). Our first hypothesis was that there is no advantage in adopting an all-out start strategy compared with a self-paced strategy characterized by a spontaneous fast-start and a final sprint. Our second hypothesis is that the all-out+5%mean trial may confer additional benefits to overall performance compared with self-paced and all-out+mean strategies.

## Material and Methods

### Participants

Twelve male cyclists participated in this study (32.3±7.2 years old, 73.3±10.7 kg, 174.5±4.7 cm). Participants had 6.2±3.3 years of experience in cycling (training frequency: 5.0±1.1 days/week and weekly training load of 124.2±45.6 min/day). All participants also participated in regional and national competitions (approximately one competition per month). Participants were classified as trained or recreationally trained cyclists, according to their maximal oxygen uptake (V̇O_2_peak: 51.8±7.0 mL·kg^-1^·min^-1^) and peak power (338±31 W) attained during a maximal incremental exercise test (26). Participants signed a written described consent form agreeing to participate in the study, which was approved by the Ethics Research Committee of the Federal University of Technology of Parana.

### Design

**Figure 1:**
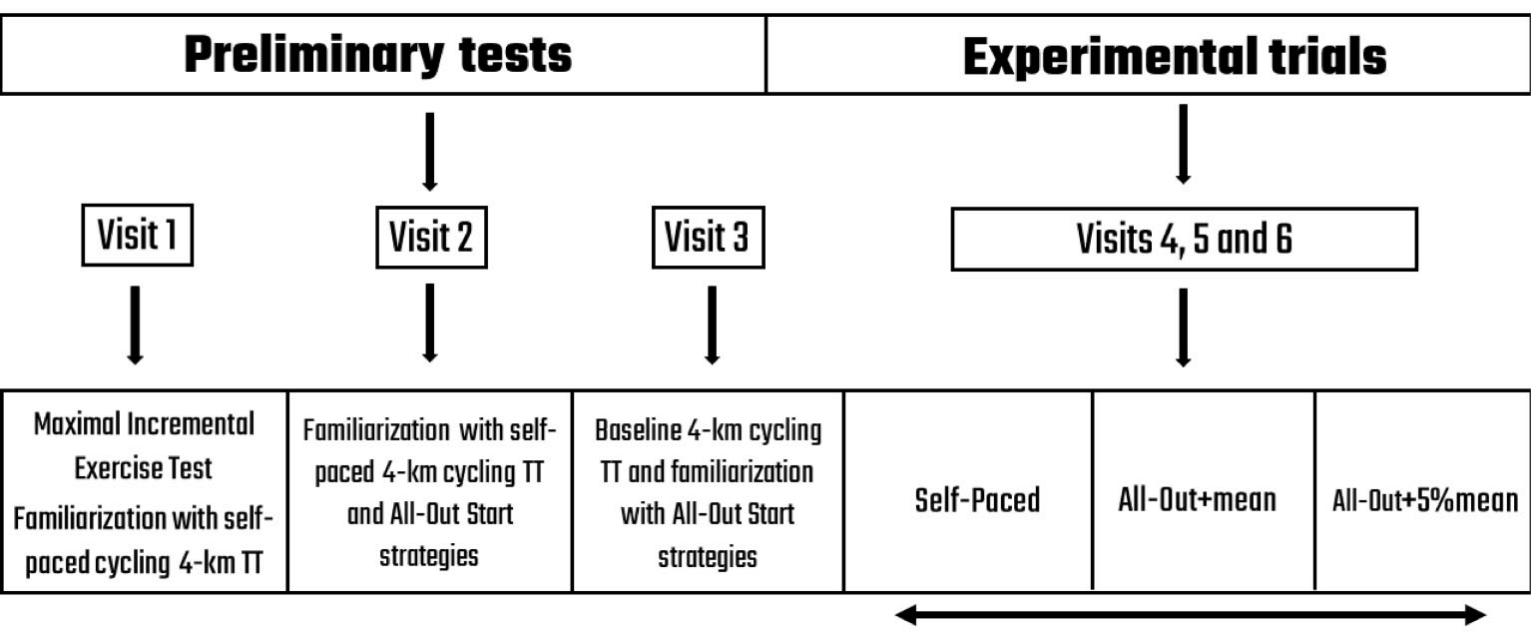
Experimental design. Bidirectional arrow indicates counterbalanced and randomized order. TT: time trial.

Participants visited the laboratory on six different occasions (there were 3 to 7 days between visits; Figure 1). On the first visit, participants performed a maximal incremental exercise test and, after a 30-min passive recovery period, a self-paced 4-km cycling time trial for familiarization. On the second visit, participants performed a second familiarization with the self-paced 4-km cycling time trial and, after a 30-min passive recovery period, practiced the all-out start strategies. On the third visit, participants performed a 4-km cycling time trial as quickly as possible, adopting their own pacing strategy. This 4-km cycling time trial is referred to as a “baseline trial”. After 30 min of passive recovery, participants practiced the all-out strategies again.

On the last three visits, participants performed in a randomized and counterbalanced order: 1) a 4-km self-paced cycling time trial; 2) a 4-km cycling time trial adopting an all-out start strategy for the first 10 s, with further maintenance at mean power of the baseline trial for the remaining first km, and a self-paced cycling thereafter (all-out+mean); and 3) a 4-km cycling time trial adopting an all-out start for the first 10-s, with further maintenance 5% above the mean power of the baseline trial for the remaining first km, and self-paced cycling thereafter (all-out+5%mean). We chose to keep a power 5% above the mean power because a preliminary pilot study indicated that this was tolerated well and did not cause premature fatigue.

All trials were performed on the participant's own bike attached to a CompuTrainer (CompuTrainer^®^ Pro, RacerMate^®^, USA). The CompuTrainer was calibrated before each trial following the recommendations from the manufacturer. The tire pressure and its pressure on the roller were checked before each trial to guarantee that no slippage occurred.

### Maximal incremental exercise test

A maximal incremental exercise test was performed to determine V̇O_2_peak and peak power. Participants warmed up at 75 W for 5 min and then the work rate was increased by 25 W every minute until exhaustion. Participants maintained a pedal cadence between 70 and 80 revolutions per minute. Exhaustion was assumed when pedal cadence dropped below 70 revolutions per minute for more than 5 s or due to voluntary disengagement. Throughout the test, pulmonary ventilation (V̇E), oxygen uptake (V̇O_2_), and carbon dioxide (V̇CO_2_) production were measured every 10-s interval via a metabolic cart (PowerLab, 40/3, ADInstruments^®^, Australia). Before each test, the metabolic cart was calibrated using ambient air and gases of known concentration (16% O_2_ and 4% CO_2_). Heart rate was continually measured using a heart rate monitor (FT1, Polar ElectroOy, Finland). The RPE was measured every stage using a 15-point Borg scale (27).

The V̇O_2_peak was determined as the highest 20-s V̇O_2_ mean during the last stage of the test. The peak power was determined as the highest power achieved during the last complete stage using the fractional time supported in the last incomplete stage multiplied by the increment rate (28).

### Experimental procedures

Participants warmed up for 5 min at 100 W with a pedal cadence between 80 and 90 revolutions per minute. Thereafter, participants performed a 4-km cycling time trial using a self-paced, all-out+mean, or all-out+5%mean strategies. The gear ratio was standardized at the beginning of each trial (52×17). During the self-paced trial, participants were free to adjust the gear once the trial had started. During the all-out+mean or all-out+5%mean strategies, participants pedaled as quickly as they could during 10 s and then maintained the target power for the remaining 1 km without switching gear. We chose to manipulate the first km of the 4-km cycling time trial because the first quarter of a short-duration time trial has been considered the most important part of the trial (6,7). The target power was visible on a monitor positioned in front of the participant. After covering the first km, participants were free to change gear. Distance and power were recorded every second via the Racermate software (CompuTrainer). The distance covered was visible on a monitor positioned in front of the participant, but they received no feedback of time or any other physiological variable. The V̇O_2_ was measured every 10 s and heart rate and RPE at the end of each km, using the same procedures described in the maximal incremental exercise test. As previously used in studies with pacing strategies (14), power and V̇O_2_ were averaged every 200 m for a more sensitive analysis of the effect of the tested pacing strategies on dependent variables.

### Statistical analysis

Data distribution was analyzed using the Shapiro-Wilk test. Once normality in data distribution was confirmed, results for performance time, mean power output, and the area under the curve of V̇O_2_ data for the first 60 s of the trial were compared between strategies using repeated measures one-way ANOVA. The same test was also used to determine if V̇O_2_peak, HRpeak, and RPEpeak of the experimental trials were different from the corresponding values of the maximal incremental exercise test. The main effect of strategy (self-pace, all-out+mean, and all-out+5%mean), distance (200 m splits), and interaction between strategy and distance on dependent variables (power, V̇O_2_, heart rate, and RPE) were verified using two-way repeated measures ANOVA. The effect size for main effects and interactions was determined using the partial eta-squared (η_p_^2^) (29). When necessary, the Duncan's *post hoc* test was used to locate differences. Performance times of the baseline trial and the self-paced trial were used to calculate the reliability of the self-paced time trial using a paired *t*-test, coefficient of variation, and typical error of measurement. The level of significance was set at P<0.05. The data are reported as means±SD.

## Results

**Figure 2:**
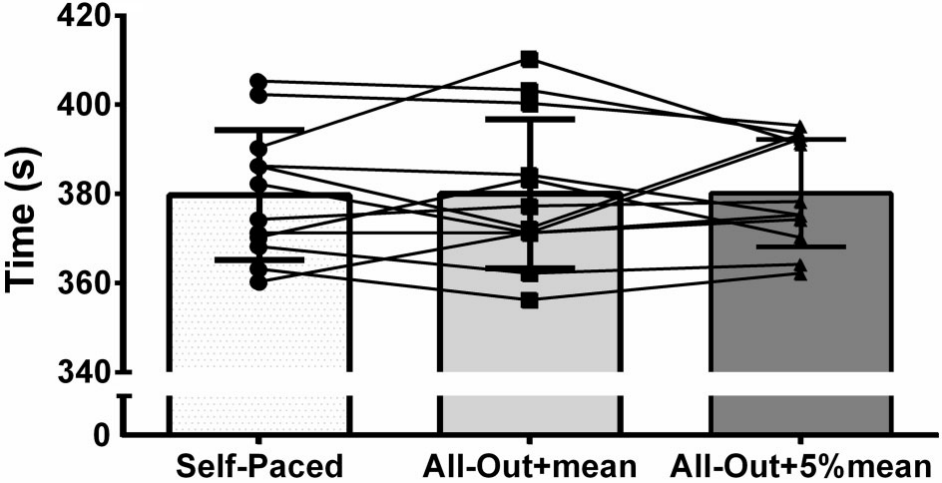
Overall performance during a 4-km cycling time trial adopting a self-paced strategy, an all-out start strategy for the first 10 s, with further maintenance at mean baseline power for the remaining first km and self-paced thereafter, and an all-out start strategy for the first 10 s, followed by a power 5% above the baseline for the remaining first km and self-paced thereafter. Data are reported as means±SD and lines are individual data.

There was no significant difference (*t*_(11)_=0.525, P=0.610) between baseline and the self-paced trial (380.3±15.5 and 379.8±14.6 s). Coefficient of variation was 0.57±0.41% and typical error of measurement was 2.64 s (0.69%). There was no significant difference (F_(2,22)_=0.010; P=0.990; η_p_^2^=0.001) between the three strategies for overall exercise performance (self-paced 379.8±14.6 s, all-out+mean 380.0±16.7 s, and all-out+5%mean 380.2±12.1 s, Figure 2). Similarly, there was no significant difference (F_(2,22)_=0.298; P=0.745; η_p_^2^=0.264) between the three strategies for mean power output (self-paced 286.3±33.5 W, all-out+mean 288.3±34.8 W, and all-out+5%mean 284.7±25.1 W).

The V̇O_2_peak and RPEpeak of the experimental trials were not different from V̇O_2_peak and RPEpeak of the maximal incremental exercise test (F_(3,21)_=0.39, P=0.76, η_p_^2^=0.05 and F_(3,33)_=0.93, P=0.44, η_p_^2^=0.08, respectively, Table 1). However, the HRpeak during the experimental trials was slightly lower than at maximal incremental exercise test (F_(3,33)_=6.74, P=0.001, η_p_^2^=0.38, Table 1).

**Table 1:** Peak oxygen uptake (V̇O_2_peak), ratings of perceived effort (RPEpeak), and heart rate (HRpeak) in the maximal incremental exercise test and 4-km cycling time trial adopting different pacing strategies.

	Maximal incremental test	Self-paced	All-out+mean	All-out+5%mean
V̇O_2_peak (L/min)	4.3±0.5	4.1±0.4	4.2±0.6	4.1±0.7
RPEpeak (arbitrary units)	19.0±1.9	18.3±1.7	18.1±2.1	17.6±2.6
HRpeak (beats/min)	182±9	176±9*	175±9*	174±8*

Data are reported as means±SD. Self-paced: pace chosen by cyclist; All-out+mean: all-out start for the first 10 s followed by maintenance at mean baseline power for the remaining first km and self-paced thereafter; All-out+5%mean: all-out start for the first 10-s followed by maintenance at 5%-higher mean baseline power for the remaining first km and self-paced thereafter. *P<0.05 compared to maximal incremental test (ANOVA).

**Figure 3:**
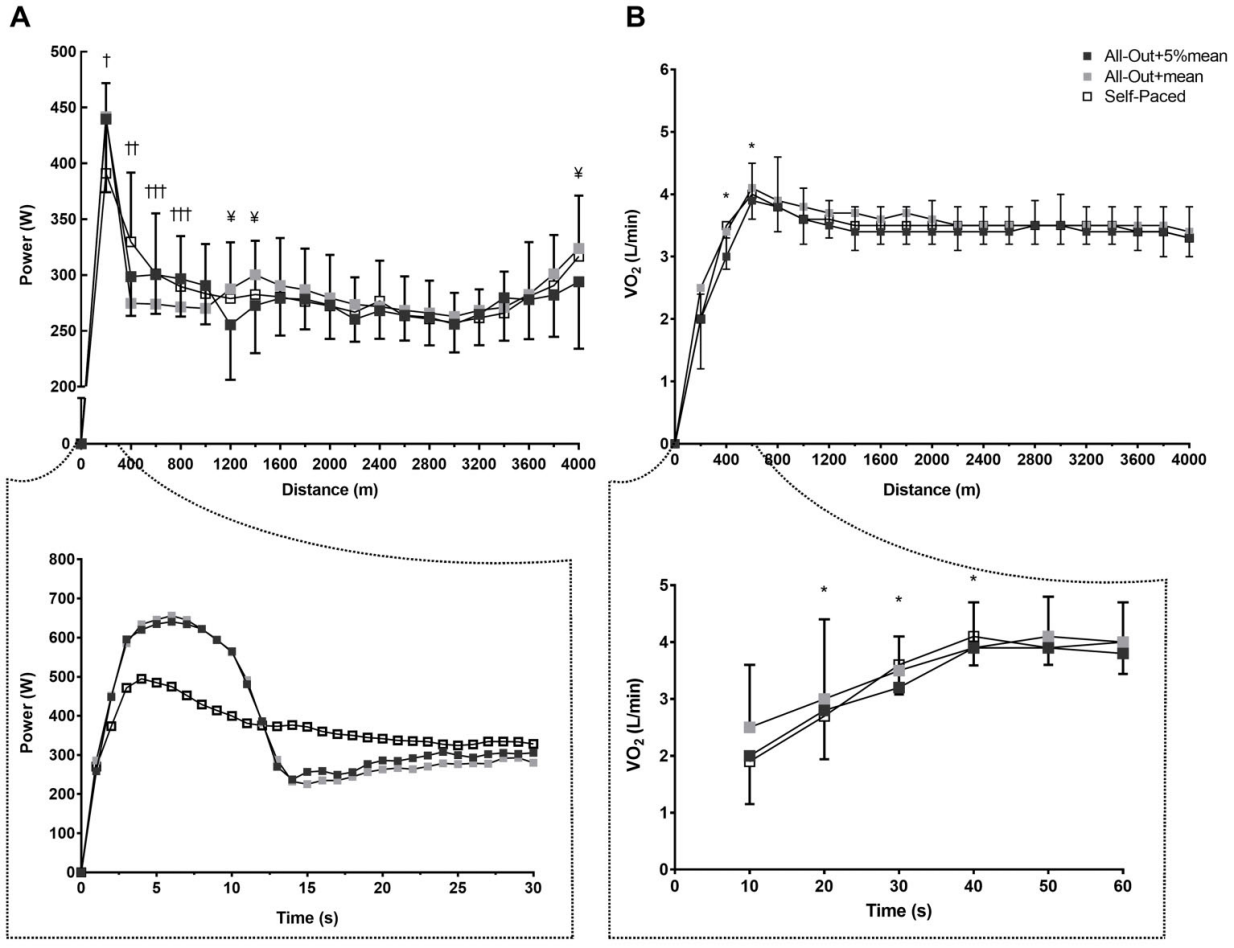
Power (upper panel **A**) and oxygen uptake (upper panel **B**) during a 4-km cycling time trial with different pacing strategies. The second-by-second mean power during the first 30 s (∼300 m) is shown in lower panel **A** (SD is omitted for better visualization). Means±SD of oxygen uptake for the first 60 s (∼700 m) are shown in lower panel **B**. ^†^P<0.05 for all-out+mean and all-out+5%mean compared to self-paced strategy. ^††^P<0.05 for the self-paced strategy compared to the all-out+mean and all-out+5%mean. ^†††^P<0.05 for the self-paced and all-out+5%mean compared to the all-out+mean strategy. ^¥^P<0.05 for the all-out+5%mean compared to the all-out+mean and self-paced strategies. *P<0.05 compared to the previous one for all pacing strategies (ANOVA).

There was an interaction between strategy and distance for power (F_(38,418)_=3.680; P=0.001; η_p_^2^=0.250, Figure 3, panel A). As expected, power at 200 m was significantly higher in the all-out+mean and all-out+5%mean strategies than in the self-paced strategy (both P=0.001), without differences between the all-out+mean and all-out+5%mean strategies (P=0.81). There was an inversion at 400 m, when power was significantly higher in the self-paced strategy than in both all-out+mean and all-out+5% mean strategies (both P=0.01). Power at 600 and 800 m was higher in the self-paced and all-out+5%mean strategies compared with the all-out+mean strategy (P<0.05).

After the first km, when cyclists were free to regulate their own pacing in all strategies, power was immediately affected in the all-out+5%mean, with a lower power at 1200 and 1400 m than in the other two strategies (P<0.05). There were, however, no differences in power between strategies from 1600 to 3400 m (P>0.05).

Final sprint in the last 200 m was impaired in the all-out+5%mean strategy. Power at 4000 m was significantly lower in the all-out+5%mean compared with the self-paced and all-out+mean strategies (P<0.05).

There was no effect of strategy and strategy versus distance interaction for V̇O_2_, RPE, and heart rate (all P>0.05). However, there was only a main effect of distance for V̇O_2_ (F_(19,133)_ =16.09; P=0.001; η_p_^2^=0.69), RPE (F_(3,33)_=66.65; P=0.001; η_p_^2^=0.85), and heart rate (F_(3,33)_ =34.12; P=0.001; η_p_^2^=0.75), suggesting that V̇O_2_, RPE, and heart rate increased similarly in all three pacing strategies as the distance progressed. Specifically, for all strategies, V̇O_2_ increased until 600 m (P<0.05) and then remained relatively stable until the end of trial (P>0.05), which corresponded to an increase of V̇O_2_ until 40 s (P<0.05) with further stabilization (Figure 3, panel B). The area under the curve for the first 60 s was also not significantly different (F_(2,14)_=0.39, P=0.684, η_p_^2^=0.05) between the strategies (self-paced: 2.88±0.35, all-out+mean: 2.96±0.63, and all-out+5%mean: 2.78±0.34 L). The RPE and heart rate increased progressively and linearly during all trials (Table 2).

**Table 2:** Ratings of perceived effort (RPE) and heart rate responses during a 4-km cycling time trial adopting different pacing strategies.

Distance	RPE (arbitrary units)			Heart Rate (beats/min)		
	Self-Paced	All-Out+mean	All-Out+5%mean	Self-Paced	All-Out+mean	All-Out+5%mean
1 km	13.1±1.7	13.3±2.6	13.4±2.2	164±10	163±8	165±10
2 km	14.8±2.2*	14.8±2.6*	14.7±2.4*	168±10*	167±10*	168±10*
3 km	16.3±2.1*	16.3±2.4*	16.3±2.2*	171±10*	171±9*	170±10*
4 km	18.3±1.7*	18.1±2.1*	17.6±2.6*	176±9*	175±9*	174±8*

Data are reported as means±SD. Self-paced: pacing chosen by cyclist; All-out+mean: all-out start for the first 10 s followed by maintenance at mean baseline power for the remaining first km and self-paced thereafter; All-out+5%mean: all-out start for the first 10 s followed by maintenance of 5%-higher mean baseline power for the remaining first km and self-paced thereafter. *P<0.05 compared to the previous km (ANOVA).

## Discussion

It has previously been reported that all-out start strategy is superior to “forced” even- or fast-start strategies (6-8). Here, we add that two different all-out start strategies were not superior to a self-paced strategy, in which a natural fast start and a final sprint occurred. Thus, the adoption of an all-out start (∼10 s), followed by either maintenance of mean power or of mean power+5% for the remaining first km does not confer a benefit to the overall performance during a 4-km cycling time trial compared to a self-regulated pacing strategy.

Overall performance was similar between the self-paced and the two all-out start strategies (Figure 2). While it seems clear that an all-out start is superior to a “forced” even- or fast-start (6-8), little is known about the superiority of an all-out start strategy compared to a self-paced strategy. In the present study, we hypothesized that the all-out+mean does not confer any gain in overall performance because we expected that the self-paced trial would be performed adopting a natural U-shaped pacing profile. A U-shaped profile is characterized by a fast start and a final sprint (14,15,18,30). A great number of studies show that cyclists naturally adopt a fast-start strategy during a 4-km cycling time trial (14,15,31). These findings suggest that any potential advantage of an all-out start strategy is abolished when compared to a self-paced strategy with a fast start. Nevertheless, a previous study demonstrated that an all-out start strategy is also superior to a fast-start strategy during a 5-min time trial (6). In that study, however, the fast-start was “forced” and initial power (first 15 s) of the all-out start strategy was twice higher than the fast-start strategy (800 *vs* 400 W). In the present study, fast start was naturally adopted and the difference in power during the first 10 s between all-out start strategies and self-paced was only 30% (∼650 *vs* ∼450 W, Figure 3, lower panel A). Interestingly, a “forced” fast start negated the final sprint (6), probably due to an increased metabolic disturbance caused by the imposed fast start. In the present study, however, the natural fast start of self-paced strategy did not impede the final sprint. These results suggested that athletes can set an “optimal” fast start during a self-paced trial, leaving some physiological reserve for a final sprint. In addition, these findings also suggested that there was no additional advantage of an all-out start strategy compared to a self-paced strategy.

We further explored whether an all-out start strategy followed by a power slightly above the mean power of the baseline trial for the remaining first quarter of the trial would improve overall performance. Although the power during the first 200 m was higher in the all-out+5%mean than in the self-paced strategy, this may have partially been compensated by a lower power at 400 m in the all-out+5%mean than in the self-paced strategy, and no differences from 600 to 1000 m (Figure 3, upper panel A). The RPE at the end of the first 1000 m was also similar between all-out+5%mean strategy and self-paced strategy (Table 2). This indicated that there were no differences in accumulated fatigue for the first km between these two pacing strategies. However, after the first km, when athletes could regulate their own pacing, the power at 1200 and 1400 m was reduced in all-out+5%mean in relation to self-paced strategy. A final sprint during the last 200 m of the trial was also impaired in the all-out+5%mean strategy (Figure 3, upper panel A). As overall performance was similar between the all-out+5%mean and self-paced strategies, these findings suggested that a potential gain from the increased initial power of the all-out+5%mean was abolished by a reduction in power immediately after the first quarter of the trial and by an incapacity to perform a final sprint. The fact that the self-paced strategy was not different from the other two all-out start strategies reinforces the assumption that cyclists can self-select the best pacing strategy to provide optimal distribution and fine-tuning power throughout a 4-km cycling time trial.

Our findings of similar overall performance regardless of the adopted pacing strategy are in accordance with the pioneer teleoanticipation model, which stipulates that the central nervous system regulates the athlete's rhythm through complex calculations based on metabolic reserve, the rate of use of these reserves, and the time remaining to complete the task to optimize performance without causing drastic disturbances to homeostasis (32). Advances in this interpretation suggest that the brain regulates pacing via different levels of awareness, in which only large homeostatic disruptions attract conscious awareness and evoke a behavioral response (20). This makes pacing regulation a behavioral expression of a continuous decision-making process, in which the interdependence of perception and action regulate exercise intensity (17). Several factors such as knowledge of the endpoint, prior experience, and external (environmental) and internal (metabolic) signals are involved in this regulation (18). This complex, interactive regulatory process might explain the abrupt decline in power at 1200 and 1400 m during the all-out+5%mean strategy (Figure 3, upper panel A). As athletes were free to change pacing after the first 1000 m, they may have reduced power to compensate for the “higher-than-expected” homeostatic perturbations during the early part of the trial; otherwise, they may not have been able to complete the task. Similarly, the all-out start for longer than 10 s would also have resulted in a large accumulation of by-products from the lactic energy system, which could compromise the conclusion of the time trial. This is also in line with the assumption that athletes continuously monitor the risk of premature exercise cessation by considering the momentary RPE and the remaining part of the race distance (33), as well as the levels of central and peripheral fatigue (25). In addition, a 4-km cycling time trial is expected to be almost entirely performed above the critical power (5), which can be assumed in the present study, as V̇O_2_peak values of the experimental trials were not different from those of the maximal incremental exercise test (Table 1). It has been demonstrated that the work completed above critical power during high-intensity cycling is invariant irrespective of the pacing strategy (forced- or self-paced), suggesting that any excessive acceleration at the beginning will need a large amount of work above critical power and must be further compensated to retain the same total work performed above critical power (34). Our findings suggested, therefore, that athletes choose optimal intensities along the trial to complete the task in the shortest possible time, without premature fatigue. It should be taken in account, however, that in a real competition, motivation and voluntary aspects might affect these choices. Further studies testing different pacing strategies in a real competition will be necessary to test this hypothesis.

In the present study, the V̇O_2_ response was not different between pacing strategies (Figure 3, panel B). The amount of V̇O_2_ consumed during the first 60 s of the trial (area under the curve) was also similar between pacing strategies. The adoption of all-out start strategies should result in greater V̇O_2_ response (6-8,12,13). However, as mentioned previously, the all-out start strategy has been compared to an even-paced strategy (3,6-8,14). Thus, the differences in power between the tested pacing strategies at the beginning of the trial may have not been great or long enough to influence V̇O_2_ response. When a fast start is already adopted during a self-paced strategy, a greater V̇O_2_ response is expected, which may reduce the differences in V̇O_2_ response between the pacing strategies. Thus, these findings might indicate that athletes optimize their V̇O_2_ response by choosing a fast start during a self-paced time trial.

The heart rate increased linearly as the distance progressed and this increase was similar in all three pacing strategies (Table 2). This is contrary to a study that demonstrated a higher mean heart rate and power output with the adoption of a fast start strategy during a 5-min cycling time trial compared with an even-paced or slow-start strategy (7). A probable explanation for the lack of difference in heart rate response between the three tested pacing strategies in the present study is that a similar heart rate response is expected when the mean power output difference between pacing strategies is lower than 5% (35,36). In our study, although power output distribution throughout the trial differed considerably between the tested pace strategies, mean power output was not significantly different. This lack of difference in mean power output might explain the similar heart rate response across the pacing strategies.

While our findings provide important insights suggesting that an all-out start strategy is not superior to a self-paced strategy, further studies adopting an all-out start strategy followed immediately by a self-paced strategy would provide more information about the benefits of an all-out start strategy. It is important to highlight that the time trials were performed without opponents. Cycling competitions consist of individual time trials, in which the cyclist competes alone, or individual and team pursuit, in which the cyclist competes simultaneously with other riders. Because the presence of other riders will influence exercise performance during time trials (37-40), our results are constrained to individual time trials.

Some limitations of the present study must be mentioned. We did not include an experimental condition with an even-paced strategy; thus, no comparison can be made between self- and even-paced strategies. We assumed, however, that a self-paced strategy with a fast start is expected to be superior to an even-paced strategy, as it has clearly been shown in multiple studies that an all-out start or a fast-start are superior to even pace (3,6-8,14). Thus, the addition of an even-paced strategy would have not changed the main conclusions of the present study. In addition, the all-out start strategies in the present study were designed to maintain a constant power for the remaining first km (25% of the trial). The power after the all-out start was fixed for the remaining first quarter of the trial to enable us to compare the results with those of previous studies that manipulated the all-out start strategy (6,7).

In conclusion, an all-out start strategy, followed by maintenance at mean power or even a 5%-higher mean power for the remaining first quarter of the trial did not confer additional benefits to overall performance of trained/recreationally trained cyclists compared to a self-paced strategy with a natural fast start. Findings of the present study have practical implications, suggesting that forced pacing conferred no additional benefits to performance; thus, cyclists should be free to choose their own pacing strategy during a 4-km cycling time trial. Nevertheless, as the response to a given pace strategy obviously had an individual component (see Figure 2), coaches are encouraged to determine which pace strategy would be better for a given athlete.
